# Enhancing perceptual and attentional skills requires common demands between the action video games and transfer tasks

**DOI:** 10.3389/fpsyg.2015.00113

**Published:** 2015-02-10

**Authors:** Adam C. Oei, Michael D. Patterson

**Affiliations:** Division of Psychology, School of Humanities and Social Sciences, Nanyang Technological UniversitySingapore, Singapore

**Keywords:** action video games, training, transfer, selective attention

## Abstract

Despite increasing evidence that shows action video game play improves perceptual and cognitive skills, the mechanisms of transfer are not well-understood. In line with previous work, we suggest that transfer is dependent upon common demands between the game and transfer task. In the current study, participants played one of four action games with varying speed, visual, and attentional demands for 20 h. We examined whether training enhanced performance for attentional blink, selective attention, attending to multiple items, visual search and auditory detection. Non-gamers who played the game (Modern Combat) with the highest demands showed transfer to tasks of attentional blink and attending to multiple items. The game (MGS Touch) with fewer attentional demands also decreased attentional blink, but to a lesser degree. Other games failed to show transfer, despite having many action game characteristics but at a reduced intensity. The results support the common demands hypothesis.

## Introduction

Recent research from multiple independent laboratories has shown video game play improves many different cognitive and perceptual skills (Greenfield et al., 1994; Green and Bavelier, 2003; Achtman et al., 2008; Strobach et al., 2012; Oei and Patterson, 2013, 2014b). Among video game genres, action video games are by far the most studied and have demonstrated the most varied transfer to laboratory tasks measuring perception and attention (but see Boot et al., 2011; Kristjánsson, 2013).

However, what constitutes an action video game is still arbitrary and open to debate. Within the “action” video game genre, there exist a wide variety of different game types often with different perceptual and cognitive demands (Latham et al., 2013). While the majority of studies have used first person and third person shooters (e.g., Green and Bavelier, 2003; Cohen et al., 2007; Oei and Patterson, 2013), others have also included racing games in this category (Wu and Spence, 2013). Nevertheless, games that are classified as action types have several characteristics in common that may be important for training related transfer. These include unpredictability, intense speed, and requirements to track and switch attention between multiple objects and locations (often beyond-capacity) while ignoring distractors (Achtman et al., 2008; Green et al., 2010a; Hubert-Wallander et al., 2011). These cognitive demands contrast with other genres such as puzzle (e.g., Tetris) or card games (e.g., Solitaire) that are predictable and do not increase the number of items to be tracked beyond capacity (Achtman et al., 2008). Moreover, while these genres may sometimes require speeded responses, they are unlikely to require as complex, flexible, and consistently high visuomotor coordination as required in action games. In other words, for the non-action genres, typically the gamer may need to improve speed of response, or strategy, but not increase working memory or attentional capacity.

Despite the wealth of evidence documenting the benefits of action video game play, the mechanisms of training-related transfer are not well-understood. Transfer to different perceptual and attentional measures may be due to the action video games having several separate demands in common with laboratory tasks that measure attention and perception (Oei and Patterson, 2014a). Another explanation to account for the many kinds of transfer is that the differences are all due to a single more general level of improvement, which then aids performance in all of these tasks. One proposal of general training-related transfer is that action video gamers improve in probabilistic inference, or “learning to learn.” As a result of training, action video game trainees become more effective in using evidence from repeated presentations of a task to guide their decision-making and allocation of cognitive resources (Green et al., 2010b; Bavelier et al., 2012). Notably, a recent study showed that experienced action video game players made improved use of perceptual templates in an orientation identification task. Importantly, although action video game players and non-players performed equivalently in the task initially, only action gamers showed improved learning of the task-relevant statistics that aided their performance (Bejjanki et al., 2014).

Nevertheless, a few key questions remain. First, it is unclear whether this “learning to learn” proposal is action video game specific or whether it can also predict learning and transfer from other video games or other learning tasks. That is, would other forms of training also result in improvements in statistical learning? Second is whether there are limits to transfer with regards to video game training. That is, what tasks can or cannot be improved from video game training? Third, empirical evidence is needed to determine whether probabilistic inference can indeed account for improvements seen across many tasks in different video game training studies (see Oei and Patterson, 2014a). In most of the previous experiments, action gamers showed enhanced performance on tasks without any training period needed. These indicated no requirement “to learn” the task since they were already better at it when they first encountered the task, or indicated that they could learn new tasks very quickly within the testing period.

Oei and Patterson (2014a) recently argued that transfer is specific and depends on similarities between the trained video game and the laboratory task. This is related to the theory of identical elements (Thorndike and Woodworth, 1901). Note that we do not claim that tasks must be exactly alike for transfer to occur. There may be some very specific learned properties of the game, such as which key to press to fire a gun, but there may also be higher level more abstract procedures that are developed during the game that may allow transfer from the game to the behavioral measure (for one approach to explaining transfer of procedures, see Taatgen, 2013). Briefly, Taatgen (2013) argued that skills required to perform a task can be broken down into “primitive information processing elements (PRIMs)” of which some are task-specific and some are general. If two tasks share overlapping elements, those learned from Task 1 can be applied in Task 2, producing transfer.

Action video games contain several highly similar demands to many transfer tasks tested in the laboratory in training studies. These include multiple object tracking (Green and Bavelier, 2004, 2006b; Oei and Patterson, 2013), peripheral target detection (Green and Bavelier, 2003; Feng et al., 2007), and rapid attentional switching (Green and Bavelier, 2003; Cohen et al., 2007; Oei and Patterson, 2013). According to this “common demands” theory (Oei and Patterson, 2014a), each of these abilities is trained separately by the action video game, and it should be possible to find action video games that contain only a subset of these demands. Thus, transfer should only be seen to tasks that share common demands.

One advantage of the command demands theory is that it can explain transfer from playing non-action video games. Oei and Patterson (2013) extended previous findings from action game studies by showing transfer to tasks that shared common demands with trained non-action games. Specifically, those trained for 20-h using a Hidden Object search game and a Match-3 game that demanded searching for appropriate matches improved in visual search speed and accuracy even when the transfer task superficially differed from the games. Note that sometimes the transfer can be extremely specific. Tetris training resulted in mental rotation enhancements that involved Tetris-like shapes but not to mental rotation in non-Tetris shapes (Sims and Mayer, 2002; Boot et al., 2008)^1^. These specific transfer effects from action and non-action games thus support the contention of transfer dependent upon common demands rather than a general transfer mechanism.

Incidentally, research outside the field of video game training supports the view of specific transfer. First, transfer is more likely when training and transfer tasks share overlapping neural demands (Dahlin et al., 2008). Second, although evidence is still being tallied, working memory training appears to transfer only to working memory measures but transfer to fluid intelligence appear more contentious (Harrison et al., 2013; Melby-Lervåg and Hulme, 2013; Redick et al., 2013) (but see Jaeggi et al., 2008).

The current study is designed with three main aims in mind. First is to show that not all action games bring about similar transfer effects. Second is to demonstrate empirically that transfer depends on common demands between the transfer task and the trained game. Third, the design of the current study allows the testing of each of the characteristics that were argued by Achtman et al. (2008) to be important for action video game training related transfer (speed, multiple object tracking, selective attention, and attentional switch). Specifically, Achtman et al. (2008) argued that these distinguishing features in action video games place heavy demands on quickly directing visual attention in central and peripheral vision for multiple frequent and unpredictable events, leading to possible enhancement of perceptual and attentional skills.

The action video games chosen for this study have demands similar to the laboratory tasks we employed. These tasks include measures of attentional blink, attending to multiple items and selective attention. One game we chose is a fast-paced first-person shooter identical to that in Oei and Patterson (2013) (Modern Combat). Playing this game for 20-h led to transfer specifically to the attentional blink, the ability to attend to multiple objects simultaneously and selective attention tasks (Oei and Patterson, 2013). It is most similar to action games used in previous studies. The other action video games we chose are similar to Modern Combat, but have progressively fewer demands in the aforementioned areas. Two third-person shooters, MGS Touch and Super Sniper do not have demands to attend to multiple objects simultaneously. MGS Touch has faster demands in speed and the need to switch attention rapidly compared to Super Sniper. The last game, Deer Hunter has the fewest demands in the above areas and was chosen specifically for its slow speed, and limited attentional demands. The only similarity to the Modern Combat game is that it is a shooter game with a first-person view (see Table 1 for a comparison of these demands).

**Table 1:** **Summary of hypothesized demands of the videogames and cognitive tasks used**.

**Hypothesized game demands**					
**Training game**	**Speed**	**Attentional switch**	**Multiple object tracking**	**Selective attention**	**Visual search**
Modern combat	High	High	High	High	Low
MGS Touch	Moderate	Moderate	Low	Moderate	Low
Super sniper	Moderate	None	None	Low	Moderate
Deer hunter	None	None	None	Very low	Very low

Given the game demands and previous results, we expected Modern Combat training to reduce attentional blink, as well as a improve accuracy on a filter task that required selective attention and the ability to attend to multiple items in parallel. Although MGS Touch has some demands to switch attention rapidly between targets, these demands are not as intense as Modern Combat. Specifically, while there is a need to switch between targets, players have more time to do so as the enemies do not engage the players immediately upon appearing. Therefore, we will be able to determine whether these weaker demands are sufficient to cause a transfer to attentional blink, selective attention and attention to multiple items. Since, Super Sniper and Deer Hunter do not contain switching, tracking, or speed demands matched with the transfer tasks, we expected no transfer to the aforementioned tasks.

In addition to the aforementioned tasks, we included a visual search task and an auditory signal detection task. Visual search enhancements have been demonstrated following action video game training (Hubert-Wallander et al., 2011; Wu and Spence, 2013). However, despite showing visual search enhancements following a hidden object game and a Match-3 game training, Oei and Patterson (2013) failed to find visual search enhancements following training in the action video game. It is noteworthy that the visual search task in Oei and Patterson (2013) was a dual task that included a working memory task. One possibility may be that the presence of the working memory task may have masked any transfer effect. Hence, we included a single-task visual search task, first to replicate previous results, and second, to determine if transfer could occur even when the action videogames lacked an intensive visual search demand.

Previous research has also shown enhancement of target detection amidst noise following action video game training (Green and Bavelier, 2003; Oei and Patterson, 2013). However, it is unclear if the improvement as a result of action videogame training represented a general modality free attentional improvement or a specific improvement to visual attention, although a cross-sectional study suggests potential benefits to auditory attention (Green et al., 2010b). Here, we included an auditory detection task to further test the specificity of transfer. If transfer following training were specific to visual skills, we would not expect a transfer to the auditory domain. On the contrary, if improvements were a general modality free probabilistic learning attentional enhancement, there would be training-related improvements to auditory detection as well.

## Methods

### Participants

Fifty-five (29 males) undergraduates (*M*_*age*_ = 21.78, *SD* = 1.76) from Nanyang Technological University (NTU) participated for course credits and S$50 cash reimbursement. They were randomly assigned to one of four videogame training groups (see Supplementary Material for breakdown of each training group). All participants were self-reported to be non-videogame players based on the criteria that they played videogames, on average, less than 1 h per week over the past year. All participants provided written consent for participation. This study received approval and conducted in accordance to the ethical guidelines prescribed by the NTU Institutional Review Board.

### Materials

All games were played via participants' personal iPhone/iPod Touch (Apple Inc.). Interaction with the game was via a touch-sensitive interface measuring 3.5 inches diagonally.

### Training games

#### Modern Combat: Sandstorm (Gameloft^®^)

Modern Combat is a fast paced first person shooter. In this game, players controlled an in-game avatar as part of a special operations team in a war zone. Players were required to navigate hostile enemy territory and had to meet objectives such as deactivating enemy equipment. Throughout the game, multiple enemies appeared unpredictably. Therefore, to ensure survival in the game, players had to shoot at enemies as they appeared. Players controlled the game via virtual joysticks on screen and fired their weapons by touching a designated area on the screen. There were 10 levels in total and each level saw an increase in difficulty level. Each level was unlocked when a player completed a preceding level. Accordingly, this game fulfills the properties argued by Achtman et al. (2008) that are likely to lead to transfer to various perceptual and cognitive skill (unpredictability, intense speed, simultaneous tracking of multiple moving objects in central and peripheral vision). Previous research indicates that training in this game reduced attentional blink, and improved selective attention (Oei and Patterson, 2013).

#### Metal Gear Solid (MGS) Touch

This is a third person shooter where the game character controlled by the player remained stationery with only the ability to move left, right, or duck under a cover. The objective of the game was to rapidly fire at enemies that appeared at various locations. However, unlike Modern Combat, the enemies appeared sequentially rather than all at once. These enemies stayed visible for roughly 5000 ms before they fired at the player and then disappeared. Hence, there was less demand to allocate attention across several targets at once and engage an enemy immediately after shooting at another enemy. There were however, “lures” that the player had to avoid shooting at which if engaged, incurred a penalty. Players were allowed to “take cover” behind a bunker to avoid enemy attacks. However, doing so prevented players from shooting at the enemies.

#### Super Sniper

In this game, players controlled the “cross-hair” of a sniper scope to look for enemies to shoot. Enemies were located at various locations and were easily spotted. Enemies did not fire at the player unless the player pointed their scope at the enemy. The enemies were highly salient and did not engage the player immediately when spotted. A level was complete when all enemies were killed.

#### Deer Hunter

In this game, participants played as a hunter and searched for animals to fire at. Unlike the other games here, there was little speed demand. A player was required to aim a scope and fire at an animal. Points were awarded for a successful kill. If the player missed, the animal ran away and the player had to look for another animal. There was little need to filter out distractors or attend to several items simultaneously.

#### Differences between the training games

As mentioned previously, the games chosen for training here vary in demands on speed, selective attention, multiple object tracking, and visual search (see Table 1 for a summary). As these differences are arbitrarily defined, it is difficult to provide quantitative metrics of the differences. However, some distinct differences can be stated. We make some comparisons based on gameplay at the lowest level of each game (level 1).

***Differences in speed***. The reaction time to engage the targets in Modern Combat is of utmost importance because the enemies engage the player immediately upon onset. This is unlike MGS Touch, where the target does not fire at the player until ~5000 ms after onset. For Super Sniper, the enemy will engage the player only after the player engages it. For Deer Hunter, the target will not engage the player. Hence, only Modern Combat has an intense speed requirement.

***Differences in multiple object tracking***. In terms of tracking multiple objects in Modern Combat, as many as 4 enemies engage the player at once (although the number of simultaneously appearing enemies increase as the levels progress. In contrast, enemies in MGS Touch appear one at a time and the player has approximately 5000 ms to aim and fire at the enemy. If the player chooses to ignore the enemy by hiding behind the bunker, it disappears after firing at the player. For Super Sniper, the player engages one enemy at a time and unlike Modern Combat and MGS Touch, the enemy does not appear until the player points the sniper scope at the enemy. For Deer Hunter, there is only one target per screen and the target is largely stationary unless the player fires at it and misses.

***Differences in attentional switch***. Due to many targets appearing at once or in quick succession, Modern Combat has the highest demands to switch attention rapidly. The other games either allow more time to switch attention (MGS Touch), are time-negligible (Super Sniper) or only contain one target (Deer Hunter).

***Differences in selective attention***. In Modern Combat, the player is part of a team of soldiers controlled by the game AI. Therefore, attention must be directed toward only enemies firing at the player and not at the friendly soldiers. Furthermore, the environment contains several items that can be shot at which further act as sources of distraction. In contrast, the other games do not contain distractors with the possible exception of MGS Touch, which occasionally has surrendering combatants with raised hands surrounded by green making them relatively easy to inhibit.

***Differences in visual search***. Comparatively, search in Super Sniper may be most demanding. In Super Sniper, a player has to search a building for targets. However, locating a target is untimed and there are no distractors interfering with search. The other gams have fewer demands for search. In MGS Touch, the player has to locate sudden onset, salient targets, but is allowed approximately 5000 ms after onset to locate enemy. In Deer Hunter, the presence of an animal is obvious because of the sparse background. Similarly, in Modern Combat, an enemy firing at a player is also tends to “pop-out.” Hence, in these instances, any “search” is likely to be parallel in nature and is much less demanding than Super Sniper.

It is important to note however, that the differences between the games are not limited to these demands only, but likely include several other demands and characteristics. However, for parsimony, these demands were studied, as they are similar to the transfer tasks that chosen here, and thus are likely to have the greatest transfer effects.

### Transfer tasks

All experimental tasks were conducted using E-Prime 2.0 (Release candidate: 2.08.90; Psychology Software Tools, Inc., Pittsburgh, PA, www.pstnet.com). Stimuli were presented via a 19-inch LCD monitor from a distance of about 60 cm. Responses were made using standard QWERTY keyboards. In all, participants performed four computerized cognitive tasks and no other tasks were performed.

#### Attentional blink

The attentional blink task is identical to a previous study (Oei and Patterson, 2013). Participants were instructed to view a rapid stream of black letters presented sequentially in a central location on the screen subtending 0.48° of visual angle against a gray background. Presentation duration of each letter was 15 ms with a 85 ms inter-stimulus interval (ISI). Participants were instructed to monitor the stream of letters and identify a white (Target 1) letter presented amongst the black letters. Participants were also instructed to determine whether an X (Target 2) appeared after they had spotted Target 1. Target 2 appeared immediately after Target 1 (Lag 1), up to 7 letters after Target 1 (Lag 8) or not at all (see Oei and Patterson, 2013).

The attentional blink is characterized by the failure to detect Target 2 after successfully detecting Target 1, especially when Target 2 appeared 200–500 ms after Target 1 (Lags 2–5) (Raymond and Shapiro, 1992; Shapiro and Raymond, 1997). The dependent variable (DV) of interest here is Target 2 detection accuracy following successful Target 1 detection. In particular, our focus here is successful Target 2 detection in the 200–500 ms window (Lags 2–5). Target 2 appeared in 50% of the time. Participants performed 12 practice trials followed by 128 test trials.

#### Filter task

In this task, participants were presented with an array of red (targets) and blue rectangles (distractors) in different orientations. The rectangles were presented within an imaginary square subtending about 9.5° of visual angle. There were 10 conditions of differing amounts of targets and distractors. The number of targets ranged from 2 to 8 and the number of distractors ranged from 0 to 6 with the constraint that in each condition, the numbers of items did not exceed 8. As in Oei and Patterson (2013), we used two conditions (2 targets – 6 distractors and 8 targets – 0 distractor) in the filter task respectively as measures of visual selective attention and of the allocation of attention toward multiple targets to detect change. This is because there is a need to filter out a large number of distractors in the former and the need to keep track of eight items in parallel in the latter. There were 10 practice trials followed by 200 test trials. Orientation change occurred in 50% of the trials. Participants were instructed to press one of two keys when they spotted a change in orientation and another when no orientation change took place.

#### Visual search

**Figure 1:**
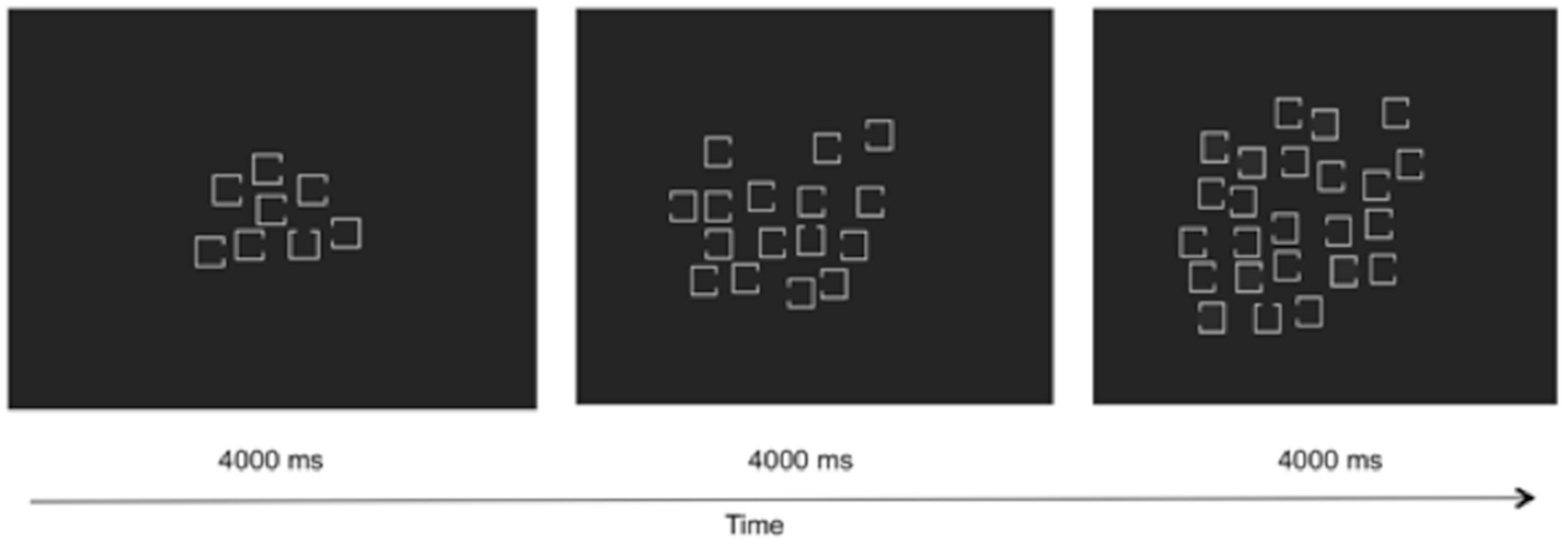
**Sample trials for visual search set sizes 8, 16, and 24**.

This task was adapted from Woodman and Luck (2004) with the modification of omitting the spatial working memory task and increasing the visual search set size to 8, 16, and 24 (see Figure 1). Depending on set size, the squares (0.45° by 0.45°) were presented within an imaginary box of 7.6° by 7.6° (set size 8), 11.31° by 11.31° (set size 16), or 12.68° by 12.68° (set size 24). There were 12 trials for each set size for a total of 36 trials. Participants were presented with squares missing a side and were instructed to make a speeded response when they saw the target—a square with a missing top or bottom. Each targeted was mapped to a different response key. Participants practiced on the task for 6 trials prior to starting the task. Each trial was presented for 4000 ms.

#### Auditory detection task

The auditory detection task is a measure of auditory attention. The task required participants to detect whether a 300 Hz beep (signal) was present in a stream of white noise. The duration of the white noise was 2000 ms while the beep occurred for 500 ms. Across all trials, the auditory stimuli (white noise and signal) were played on either the left or right sides of participants' headphones. 180 trials were presented with 120 signal-present and 60 signal-absent trials. Half the trials were played on the left and half were played to the right ear. Participants were instructed to make a speeded response by pressing the “z” key if no signal accompanied the white noise. When the signal occurred, participants were to press the “n” or “m” key to indicate the detection of the sound played on the left or right ears, respectively.

### General procedure

Participants performed all the tasks in a group in a computer lab. They were first briefed on the requirements in the study but were not explicitly told what games they were assigned to play during training. Performance of the laboratory tasks was done in a random order. Time taken to complete all laboratory tasks was about 1 h. Each participant was individually briefed upon task completion regarding the game they were to download onto their personal device (iPhone/iPod Touch). Participants were instructed to play their assigned game 5 days a week for 1 h each time (for a total of 20 h). They were also told to not exceed the amount of prescribed duration or play other games. Since participants were all non-gamers, there is little likelihood of them violating the restrictions.

After the training, they returned for the same set of computerized tasks. Participants only returned after at least 24-h after their gameplay cessation. This washout period was to control for arousal effects arising from their last session of gameplay which might in turn affect their performance at the post-test as their arousal from playing the game may induce strategy changes in the transfer tasks (cf. Nelson and Strachan, 2009). The post-training sessions were conducted in the same manner again with randomization of the transfer tasks.

At the post-training session, participants' handsets were also checked to ascertain that they downloaded the game and played it. A debriefing followed.

Participants were not informed beforehand that their devices would be checked. It was verified that all participants downloaded and played the game. Furthermore, all participants made some progress within the game they were assigned. These, taken together with their self-report of daily playing time are indicative of their fidelity to the prescribed training regime.

## Results

### Attentional blink (AB)

First, a within subjects ANOVA was conducted to determine if the attentional blink task yielded the classic attentional blink effect. There was a significant effect of lag, Wilks λ = 0.39, *F*_(6, 46)_ = 4.99, *p* = 0.001. Follow-up paired *t*-tests indicate that overall, accuracy at lag 2 was significantly lower than all other lags (all *p*s < 0.019) with the exception of lag 3 (*p* = 0.091). Accuracy at lag 3 was also lower than all others (all *p*s < 0.012) with the exception of lags 2 and 6 (*p* = 0.18). These findings are indicative of the attentional blink effect.

As the AB effect is limited to T2 detection within 200–500 ms (lags 2–5) following successful T1 detection (Raymond and Shapiro, 1992; Shapiro and Raymond, 1997), we report only results within these lags.

To ensure that each group started out having similar T2 detection accuracy performance prior to training, a 4 (training group) × 4 (AB Lags 2–5) mixed ANOVA was conducted for the lags of interest (Lags 2–5). Aside from a main effect of lag, Wilks λ = 0.75, *F*_(3, 49)_ = 5.58, *p* = 0.002, no interactions between lag and group was found, nor were there any between subjects effects (*p*s > 0.35). These results indicate that the groups had equivalent performance across these lags prior to training (see Table 3 in Supplementary Material for T2 detection accuracy for each group for AB lags 2–5). T1 detection accuracy was generally high (*M*_T1 accuracy_ = 0.977, *SD* = 0.026) and also similar between groups, *F*_(3, 51)_ = 1.21, *p* = 0.31.

A 4 (AB lags 2–5) × 4 (training groups) × 2 (time: pre and post-training) mixed ANOVA was conducted to evaluate for training gains in T2 detection. The within subjects factors were lags 2–5 and time while the between subjects factor was the training group with four levels. There was a significant main effect of lag, *F*_(3, 153)_ = 14.96, *MSE* = 0.04, *p* < 0.001 and time, *F*_(1, 51)_ = 39.09, *MSE* = 0.06, *p* < 0.001. This was qualified by a group × time interaction, *F*_(3, 51)_ = 2.79, *MSE* = 0.06, *p* = 0.05. The group × lag interaction was also statistically significant, *F*_(9, 153)_ = 2.46, *MSE* = 0.04, *p* = 0.012. All other main effect and interactions failed to reach statistical significance.

Paired *t*-tests were conducted to follow-up the significant interactions above and to determine which group improved in the AB lags of interest following training. The paired *t*-tests revealed that the improvement by the Modern Combat group in accuracies for lag 2 [*t*_(13)_ = 3.70, *p* = 0.003, Cohen's *d* = 1.61], lag 3 [*t*_(13)_ = 3.61, *p* = 0.003, Cohen's *d* = 1.43], lag 4 [*t*_(13)_ = 2.43, *p* = 0.03, Cohen's *d* = 0.95], and lag 5 [*t*_(13)_ = 2.43, *p* = 0.03, Cohen's *d* = 1.00] were statistically significant.

**Figure 2:**
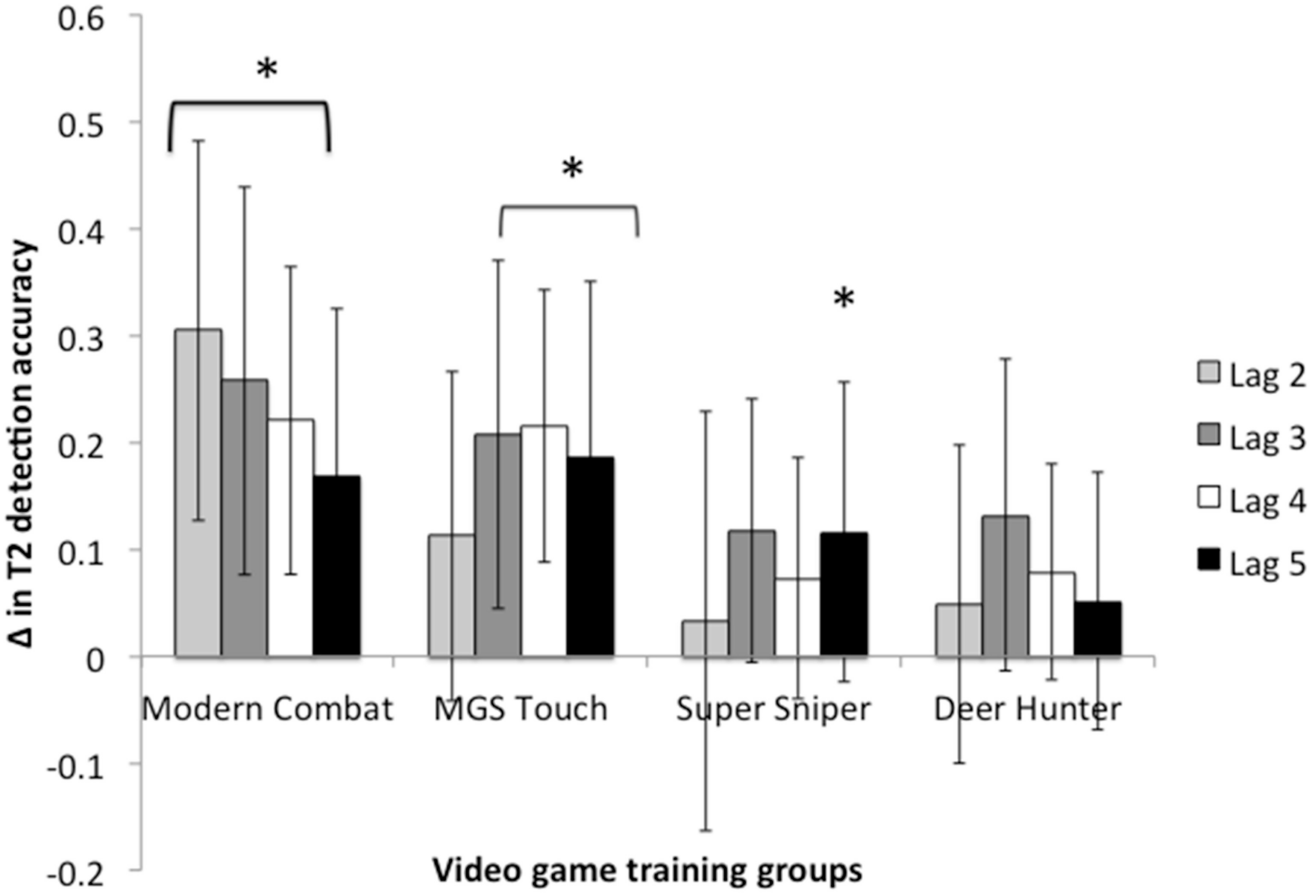
**Changes (Δ) in T2 detection accuracy rate for each training group following training**. Asterisks denote statistical significance at *p* < 0.05. Error bars denote 95% CI.

There were also statistically significant improvements in lag 3 [*t*_(13)_ = 2.75, *p* = 0.016, Cohen's *d* = 1.46], lag 4 [*t*_(13)_ = 3.79, *p* = 0.002, Cohen's *d* = 1.76], and lag 5 [*t*_(13)_ = 2.75, *p* = 0.017, Cohen's *d* = 1.22] for the MGS Touch group. In contrast, improvements in accuracy rate for lag 2 failed to reach statistical significance (*p* = 0.20). Paired samples *t*-test conducted for the four lags for the other training groups revealed that aside from the Super Sniper group's improvement in lag 5 reaching statistical significance (*p* = 0.027, Cohen's *d* = 1.05), no other improvements were seen (Figure 2).

While the above analyses tested whether training resulted in greater improvements in AB, further analyses were also conducted to determine if training resulted in superior AB performance post-training. A One-Way ANOVA with contrast analyses conducted revealed that Modern Combat group outperformed all other groups at post-training for lag 2 (*p* = 0.001) only. The groups did not differ in T1 accuracy rate. This suggests that there were no strategic differences between the groups in the approach to the AB task. Finally, we also evaluated whether T1 detection accuracy changed from pre to post training and whether the changes differed between groups. Although changes in T1 accuracy were statistically significant following training, *F*_(1, 51)_ = 10.29, *p* = 0.002, the gains were not different between groups, *F*_(3, 51)_ = 0.25, *p* = 0.86.

### Filter task

The DVs in the task were calculated as a sensitivity index, *d*' for each of the 10 conditions. This was derived from correct change detections and false alarms. The formula used to derive the *d*' statistic is based on (Tanner and Swets, 1954).

Although two conditions (2 target 6 distractors condition and the 8 target 0 distractor condition) were of primary interest here, we analyzed all 10 conditions to determine if training improved any of the conditions (see Table 4 in Supplementary Material for *d*' means for each condition by training group). First, we entered all 10 filter task conditions into a 4 (training group) × 10 (filter task conditions) mixed ANOVA to determine if pre-training differences existed between groups. The mixed ANOVA showed no group × condition interaction, *F*_(27, 459)_ = 1.26, *MSE* = 0.31, *p* = 0.17. These results indicate that prior to testing, detection sensitivity (*d*') was equivalent between the groups. However, there was a main effect of condition, *F*_(9, 459)_ = 57.98, *MSE* = 0.31, *p* < 0.001.

A 2 (time) × 4 (training groups) × 10 (conditions) ANOVA was conducted to determine if any of the groups improved performance in the filter task following training. The mixed ANOVA revealed a main effect of condition, *F*_(9, 459)_ = 99.12, *MSE* = 0.38*, p* < 0.001. This was qualified by a three-way time × condition × training group interaction, *F*_(27, 459)_ = 1.56, *p* = 0.037.

**Figure 3:**
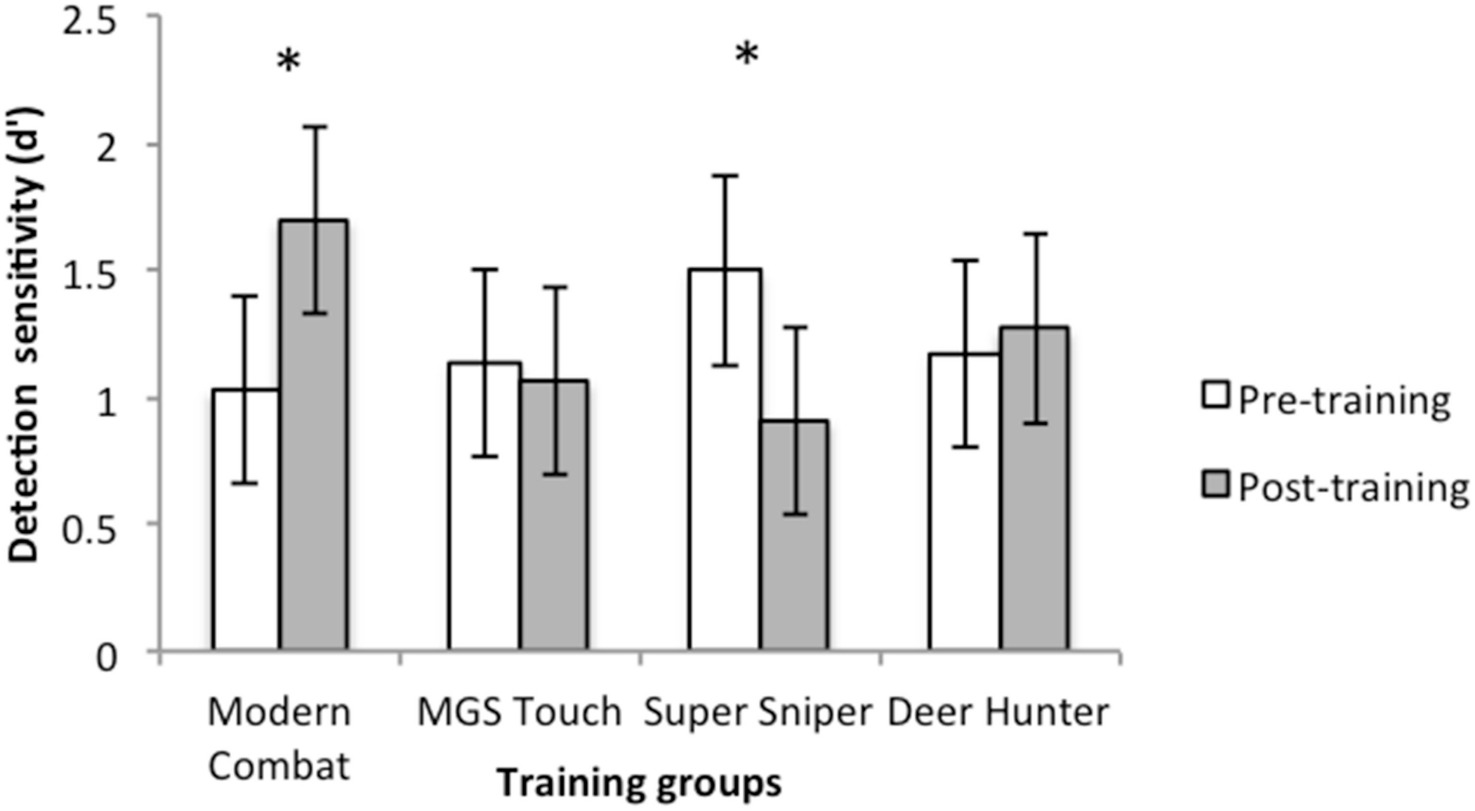
**Detection sensitivity (*d*′) for 8 target 0 distractor condition from pre to post-training for each training group**. Asterisk denote statistical significant change at *p* < 0.05. Error bars denote 95% CI.

Paired *t*-tests were conducted for each group to determine if performance improved as a result of training. For the Modern Combat group, the improvement in *d*' from pre (*M* = 2.70, *SD* = 0.56) to post training (*M* = 3.02, *SD* = 0.28) for the 2 target 6 distractor condition approached significance, *t*_(13)_ = 2.08, *p* = 0.058, Cohen's *d* = 0.84. Conversely, there was a significant improvement in the 8 target 0 distractor condition, *t*_(13)_ = 2.61, *p* = 0.022, Cohen's *d* = 0.99. With the exception of an improvement by the Deer Hunter group in the 2 target 4 distractor condition (*p* = 0.002) and a performance deterioration by the *Super Sniper* group in the 8 target 0 distractor condition (*p* = 0.007), no other groups showed performance changes following training (all *p*s > 0.05) in any condition. These results indicate that only the Modern Combat group improved in the ability to apprehend multiple objects simultaneously (see Figure 3). Furthermore, contrast analyses show that post-training sensitivity (*d*′) was greater in the Modern Combat group relative to the other groups (*p* = 0.013).

### Visual search accuracy

For visual search, the DVs are RT for correct detections and accuracy rates. A 2 (time: pre and post training) × 3 (set size) × 4 (training groups) ANOVA was then conducted to determine if training resulted in accuracy improvements for any of the set sizes. There was a significant main effect of time, *F*_(1, 50)_ = 30.16, *MSE* = 0.004, *p* < 0.001, and set size, *F*_(2, 100)_ = 115.61, *MSE* = 0.004, *p* < 0.001. This was qualified by a time × set size interaction, *F*_(2, 100)_ = 14.58, *MSE* = 0.003, *p* < 0.001. All other interactions failed to reach statistical significance. This suggests that the groups did not differ in the magnitude of improvements in visual search accuracy. These results also indicate the lack of pre-training differences between the groups.

### Visual search RT

A 2 (time: pre and post training) × 3 (set size) × 4 (training groups) ANOVA was conducted to determine if performance improved following training. There was a significant time main effect, *F*_(1, 50)_ = 26.31, *MSE* = 32967.26, *p* < 0.001. There was also a significant main effect of set size, *F*_(2, 100)_ = 588.37, *MSE* = 16008.71, *p* < 0.001. All interactions failed to reach statistical significance. These results suggest that the groups had equivalent search RTs prior to training and also that improvements did not differ between groups (see Figure 4 in Supplementary Material for pre and post-training data for visual search RT for each group).

### Auditory detection task detection accuracy

A 2 (time) × 2 (ear: left vs. right) × 4 (training group) repeated-measures ANOVA was conducted to determine if there were any improvements in auditory signal detection accuracy from pre to post training. The ANOVA revealed a significant time effect, Wilks λ = 0.92, *F*_(1, 51)_ = 4.29, *p* = 0.04. However, all other main effects and interactions failed to reach statistical significance (all *p*s > 0.11). These results suggest that the improvements from pre to post-training were equivalent between the training groups. These results indicate that the groups had equivalent performance in auditory detection prior to training.

### Auditory detection task RT

A repeated-measures ANOVA was then conducted to determine if there were any improvements in auditory signal RT from pre to post training. There was a significant time effect, Wilks λ = 0.66, *F*_(1, 50)_ = 25.66, *p* < 0.001. However, all other main effects and interactions did not reach statistical significance (all *p*s > 0.31). These results suggest that the improvements from pre to post-training were equivalent between the training groups (see Figure 5 in Supplementary Material for pre and post-training RT).

## Discussion

The overarching goal of this study was to further test the proposal in Oei and Patterson (2014a) that transfer depends on common demands between the trained game and the transfer task. Thus, we compared different types of action games to define what properties must be present in an action game for different types of transfer to occur. We mainly tested the properties of action games proposed by Achtman et al. (2008), namely attentional switching, speed, selective attention and attending to multiple items. Importantly, transfer effects varied according to the demands in the game. We discuss the results based on our hypothesis of training-related gains on each task.

### Attentional blink

We predicted that playing Modern Combat would be mostly likely to improve attentional blink performance. As attentional blink requires rapid recovery from detecting Target 1 in order to detect Target 2, we reasoned that playing Modern Combat, like most fast-paced first-person shooters that place intense demands on the temporal aspect of attention, would improve the attentional blink. This hypothesis was supported and corroborated previous findings (Green and Bavelier, 2003; Oei and Patterson, 2013). However, we note previous action video game training that failed to reduce the attentional blink following 20 h of training (Boot et al., 2008). One possibility for Boot et al.'s failure to replicate could be due to participants performing each transfer task three times - before, midway and after training. Hence, it is conceivable that test-retest and practice effects may have masked any specific transfer that arose as a result of video game training.

Here, our results show that Modern Combat training improved detection of Target 2 in lags most commonly affected by the attentional blink (2–5). Furthermore, the unique improvement in T2 detection in lag 2 by the Modern Combat group is important because T2 detection in lag 2 is comparatively most affected by the attentional blink. Hence, it suggests that only a game that had the requirement to switch attention between targets at intense speeds lead to this improvement.

Interestingly, the MGS Touch group also showed significantly improved recovery from the attentional blink (in lags 3–5). However, unlike Modern Combat, there was no evidence of transfer to the lag most susceptible to the attentional blink effect, lag 2, which is consistent with the suggested difference between attentional switching demands between Modern Combat and MGS Touch. In Modern Combat, enemies appear rapidly in succession and sometimes even concurrently and fire at the player. On the other hand, in MGS Touch, although the enemies appear rapidly in succession, they do not engage the player immediately. Players are thus allowed some time to recover from attentional capture and engage an enemy before the enemy fires back at the player. Nevertheless, they still need to recover attention fast enough to create transfer that reduces attentional blink. The demands are just not fast enough to effect performance at lag 2.

In contrast, no evidence of transfer was seen in attentional blink by playing Deer Hunter and improvements were only seen in the latest lag that is susceptible to attentional blink by the Super Sniper group. These games contain little to no demands to switch attention rapidly from one target to another, with Super Sniper only having a small requirement to respond to other targets as quickly as possible before the overall timer runs out. Hence, this is consistent with the argument that attentional blink improvements are only achieved by playing games that have the need to switch attention rapidly from one target to another.

### Attention to multiple targets

We also predicted that playing Modern Combat would improve simultaneous attending of multiple objects. This hypothesis was supported. Again, common demands between Modern Combat and the task can explain the transfer effect. For the task, participants were required to attend simultaneously to multiple items in order to detect whether there were any changes in orientation. For Modern Combat, there are heavy demands to continuously monitor and attend to multiple enemies simultaneously. As mentioned, previous work has shown that playing a fast-paced first-person shooter improves multiple object tracking for moving objects (Green and Bavelier, 2006b). Here, the finding of an improved ability to attend to multiple static objects further supports previous findings (Oei and Patterson, 2013) and extends previous work (Green and Bavelier, 2006b).

In contrast, the other games tested here that did not have demands to attend to multiple items simultaneously failed to result in transfer to attending multiple targets. This further lends weight to the assertion that intense practice of a specific skill within a game is necessary for transfer to a task that shares common demands. However, it remains a question why Modern Combat training failed to improve performance in the Filter task conditions with 6 or 4 targets. We speculate that Modern Combat training exerted performance gains only when working memory or attentional loads exceeded capacity (8 target condition).

### Selective attention

We predicted that playing Modern Combat would improve in accuracy in the selective attention (2 target 6 distractor) condition relative to the other groups. The improvement for the Modern Combat group approached significance (*p* = 0.058). Hence, this hypothesis was not supported. However, several previous studies have shown an improvement in selective attention using a first-person shooter (Green and Bavelier, 2003; Oei and Patterson, 2013). The reason for the lack of a positive transfer effect is unclear but we speculate that the sample size may not provide enough power to attain statistical significance since the improvement in performance was accompanied by a large effect size (Cohen's *d* = 0.84).

### Visual search

The results in the visual search task showed no differential transfer by any of the different training groups. This is consistent with a previous work (Oei and Patterson, 2013) that showed no action game training related transfer to visual search skills, but is inconsistent with Wu and Spence (2013). A key difference in the type of games the games used in each study may account for the discordant results. Wu and Spence argued that the racing and first person shooter game used in their study contained high search demands. In contrast, the games used here have relatively few search requirements. Rather, targets often popped out and engaged the player (Modern Combat, MGS Touch, and Super Sniper) or moved on a static background (Deer Hunter). Hence, there was little opportunity to practice search skills. Previously, Oei and Patterson (2013) showed that training in a game with high search demands (Hidden Expedition) led to improvements in visual search. Hence, further replications involving an action game with high search demands may be necessary.

### Auditory detection

We failed to find different transfer effects to auditory detection in any of the trained games. This shows that there was no cross-modality transfer to auditory attention even in the fast-paced first person shooter, Modern Combat. Our preliminary suggestion is that action videogames, especially fast paced first person shooters may bring about visual-based attentional capacities (Green and Bavelier, 2003) but not transfer across modalities (but see Green et al., 2010b; Oei and Patterson, 2013). Again, this shows the specificity of transfer that is limited to that which is trained within the game, and does not support that general probabilistic learning accounts for action video game training transfer. We do note that there was a general test-retest effect on the auditory detection task. This could limit the ability to detect a change due to training (Green et al., 2014).

### Can general learning explain the transfer effects seen in the current study?

Bavelier et al. (2012) argued that action video game related transfer is likely to be general in that action video game training improves probabilistic inference so that trainees become more effective in using evidence from prior trials to guide decision making and resource allocation. Thus, action video game training would be likely to transfer across many superficially unrelated tasks.

Recently, evidence has emerged that supports this hypothesis whereby action video gamers showed improved learning of perceptual templates to judge orientations relative to non-gamers. This improvement was also seen in naïve gamers following 50 h of action video game training (Bejjanki et al., 2014). We did not specifically test learning ability and it is possible that was also improved by playing the more demanding action game because participants must adapt to new challenges in the game. However, if the “learning to learn” hypothesis is to be used to explain transfer to multiple tasks in multiple past cross-sectional studies, then the learning must have occurred extremely quickly during the testing period.

Here, our results show that training-related transfer may be restricted to games that shared common demands with the transfer tasks. Specifically, training failed to transfer across all tasks we tested, despite exclusively using action video games. Furthermore, we find that the magnitude of transfer (at least in the attentional blink task) depended upon the intensity of the demand to switch attention in the trained game. In this case, only training in Modern Combat and MGS Touch reduced attentional blink effects. Crucially however, only Modern Combat, which had the most intense demands to switch attention rapidly, led to a reduction of the blink effect on the lag most susceptible to the blink effect. These results suggest that training and transfer effects from video game training is more likely to be specific rather than general. These results corroborate a recent cross-sectional study of two types of habitual action game players compared with non-action gamers (Dobrowolski et al., 2015). Specifically, real-time strategy players showed greater accuracy for a multiple object tracking task than non-gamers, and their superior performance over first-person shooter players trended toward significance. The authors speculated that although both first person shooter and real time strategy games require tracking multiple objects during gameplay, the demands are greater during real time strategy play.

## Limitations

We wish to highlight some limitations in this work that should be taken into context when interpreting the results. First, the sample size in each group is small which may have prevented differences in performance from reaching statistical significance. For instance, we failed to replicate previous studies for an enhancement in selective attention in the Filter task as the pre-post difference approached but did not reach statistical significance (*p* = 0.058). However, effect size was still large in the predicted direction, indicating further investigation is warranted. Note also that in the analyses for changes in performance for the Filter task, a large number of comparisons were made. This could have resulted in an inflation of family-wise error rate. Hence, performance enhancements in the Filter task should be interpreted in light of this limitation.

Second, unlike previous investigations where participants played video games under supervised laboratory conditions (e.g., Green and Bavelier, 2003, 2006a; Boot et al., 2008), participants in our study instead played the game on their own hand-held devices in their preferred time and setting. This was done so that the game playing mimics how participants normally do so, thus enhancing ecological validity. However, doing so makes ensuring training fidelity difficult. Although participants self-reported that they followed the training regimen, there was no way to verify that they had really done so. Nevertheless, during post-training sessions, we checked participants gaming devices without previously informing them we would do so. Based on the levels they achieved in the games, it appears that they had spent considerable time playing their assigned game, and thus there is no reason to doubt that they played the prescribed duration. Furthermore, this method has previously been used (Oei and Patterson, 2013, 2014b), and replicated several findings in the literature that used lab-based training with full-sized computers.

Third, we chose the games here based on surface similarities in demands between the game and transfer task. Only qualitative, but not quantitative metrics were used to show similarities in task demands. However, since the games used in this study were not designed specifically for cognitive and perceptual training in mind, it is difficult to objectively “measure” the all the demands needed for gameplay. Although the approach taken here is one that explicitly describes and contrasts the different demands in these games, the comparisons and descriptions remain subjective, similar to previous research (Cohen et al., 2007; Spence and Feng, 2010). More objective characterization of actual game demands necessary for gameplay and successful transfer remains a difficulty within the field of video game training and may require more collaboration with video game designers, or careful tabulation of response and perceptual demands in each game by recording game play. Future research could thus explore different means in which to measure game and task demands so as to provide an objective way to compare them.

## Conclusion

In this study, we demonstrated that not all action videogames are similar in bringing about cognitive and perceptual changes. Different action games contain varying cognitive demands even though on the surface, they may seem highly similar (e.g., first-person shooter perspective). This study demonstrated that transfer to a cognitive task is more likely if a common underlying skill is highly practiced in a videogame and the cognitive task. Briefly, we demonstrated that transfer from training in an action video game is dependent upon the demands made on speeded response, selective attention, multiple object tracking, and attentional switching.

We close by restating the problem we highlighted at the beginning of this paper – that although there exists a large pool of evidence documenting the benefits of video game play on human cognition, the mechanisms of transfer are not well-understood. In this paper we have attempted to narrow down and specify some requirements for transfer within action games, but much work remains. Future efforts could thus be focused on uncovering possible mechanisms and creating computational models of transfer.

### Conflict of interest statement

The authors declare that the research was conducted in the absence of any commercial or financial relationships that could be construed as a potential conflict of interest.

## Footnotes

^1^ One possibility is that the training on Tetris was over-regularized so that the participants relied upon specialized strategies that then could not be transferred to more general situations. Variability may be one important ingredient to make more general transfer possible.
