# Supplementary material for: Enhancing perceptual and attentional skills requires common demands between the action video games and transfer tasks
Source: Front Psychol. 2015 Feb 10;6:113. doi: 10.3389/fpsyg.2015.00113 (PMC4322619; doi:10.3389/fpsyg.2015.00113)
Supplement: Supplementary file 1 [file DataSheet1.DOCX]

Supplementary Material

Enhancing perceptual and attentional skills requires common demands between the action video games and transfer tasks

Table 2.

| Group | Gender *n* | Age (SD) |
| --- | --- | --- |
| Modern Combat | 9 males, 5 females | 22.07 (2.02) |
| MGS Touch | 8 males, 6 females | 22.00 (2.11) |
| Super Sniper | 6 males, 8 females | 21.79 (1.21) |
| Deer Hunter | 6 males, 7 females | 21.23 (1.69) |

*Gender and mean age of participants in each group.*

*Table 3.*

Pre and post-training Attentional Blink performance for each training group

|  | Lag 2 | | Lag 3 | | Lag 4 | | Lag 5 | |
| --- | --- | --- | --- | --- | --- | --- | --- | --- |
|  | Pre training | Post training | Pre training | Post training | Pre training | Post training | Pre training | Post training |
| Modern Combat | 0.61 | 0.91 | 0.65 | 0.91 | 0.69 | 0.91 | 0.67 | 0.84 |
| MGS Touch | 0.57 | 0.68 | 0.62 | 0.83 | 0.64 | 0.86 | 0.66 | 0.84 |
| Super Sniper | 0.62 | 0.65 | 0.77 | 0.89 | 0.83 | 0.90 | 0.82 | 0.93 |
| Deer Hunter | 0.58 | 0.63 | 0.58 | 0.71 | 0.77 | 0.85 | 0.79 | 0.84 |

Table 4

*Mean detection sensitivity (d’) for all conditions in the Filter task for each training group*

| Condition | Modern Combat | | MGS Touch | | Super Sniper | | Deer Hunter | |
| --- | --- | --- | --- | --- | --- | --- | --- | --- |
|  | Pre-training *d’* | Post-training *d’* | Pre-training *d’* | Post-training *d’* | Pre-training *d’* | Post-training *d’* | Pre-training *d’* | Post-training *d’* |
| 2 targets – 0 distractor | 2.58 | 2.57 | 2.66 | 2.91 | 2.62 | 2.56 | 2.40 | 2.46 |
| 2 targets – 2 distractors | 2.80 | 2.70 | 2.58 | 2.57 | 2.75 | 2.59 | 2.50 | 2.45 |
| 2 targets – 4 distractors | 2.59 | 2.63 | 2.45 | 2.63 | 2.62 | 2.61 | 2.07 | 2.67 |
| 2 targets – 6 distractors | 2.70 | 3.03 | 2.34 | 2.50 | 2.28 | 2.52 | 2.29 | 2.65 |
| 4 targets – 0 distractor | 2.35 | 2.33 | 2.40 | 2.27 | 2.02 | 2.14 | 2.17 | 2.01 |
| 4 targets – 2 distractors | 1.75 | 2.01 | 2.09 | 1.94 | 2.04 | 1.88 | 1.63 | 1.90 |
| 4 targets – 4 distractors | 1.47 | 1.72 | 1.32 | 1.20 | 1.49 | 1.72 | 1.59 | 1.54 |
| 6 targets – 0 distractor | 1.48 | 1.41 | 1.62 | 1.58 | 1.57 | 1.40 | 1.09 | 1.63 |
| 6 targets – 2 distractors | 1.20 | 1.04 | 1.20 | 1.13 | 1.20 | 1.52 | 1.33 | 1.21 |
| 8 targets – 0 distractor | 1.03 | 1.70* | 1.13 | 1.06 | 1.49 | 0.91* | 1.17 | 1.27 |

*Note.* Asterisks denote statistically significant change in *d’* from pre-training to post-training

Figure 4. Pre and post-training RT in visual search for each training group (A = Modern Combat; B = MGS Touch; C = Super Sniper; D = Deer Hunter) as a function of set size.

Figure 5. Pre and post-training RT in auditory detection for each training group.
