# Supplementary figures and images for: Enhancing perceptual and attentional skills requires common demands between the action video games and transfer tasks
Source: Front Psychol. 2015 Feb 10;6:113. doi: 10.3389/fpsyg.2015.00113 (PMC4322619; doi:10.3389/fpsyg.2015.00113)

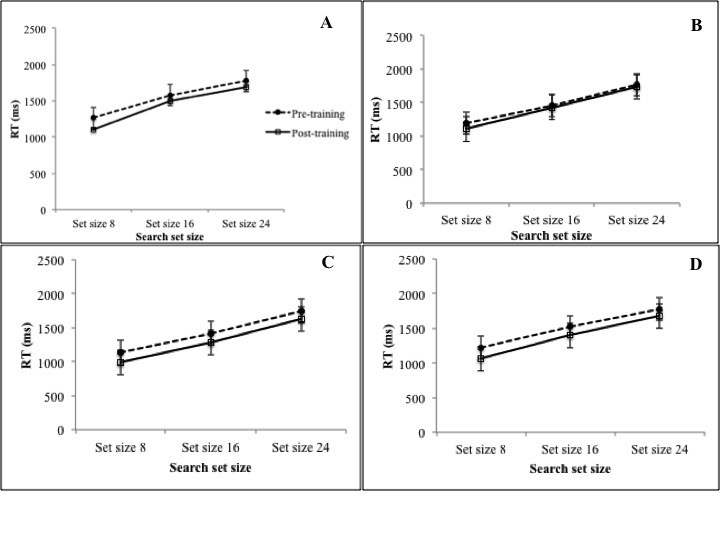

Supplement: Supplementary file 2 [file Image4.TIFF]

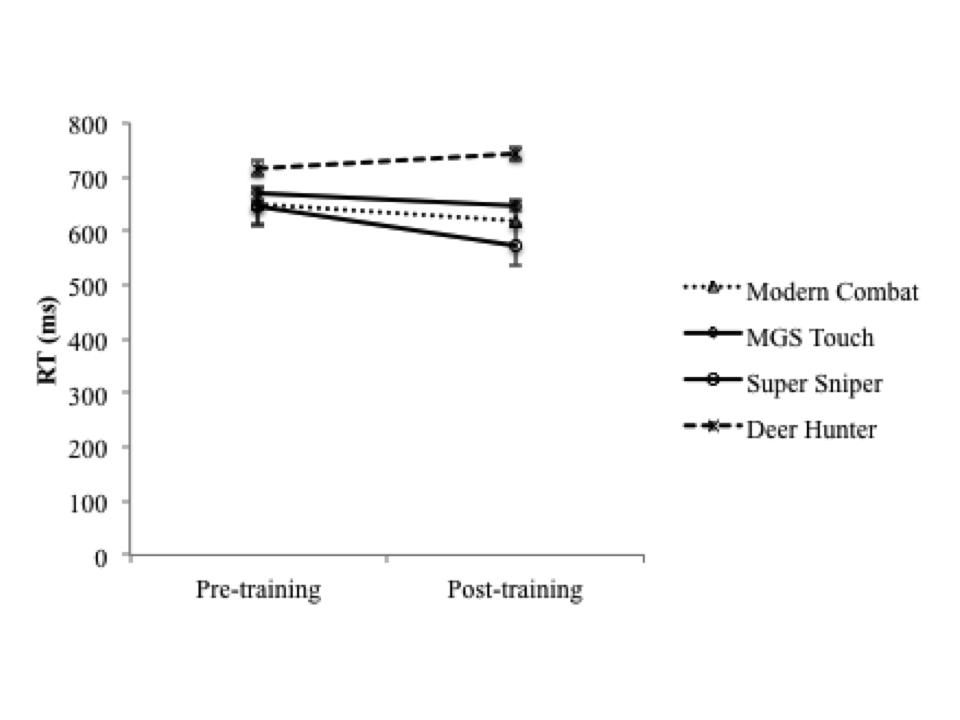

Supplement: Supplementary file 3 [file Image5.TIF]
